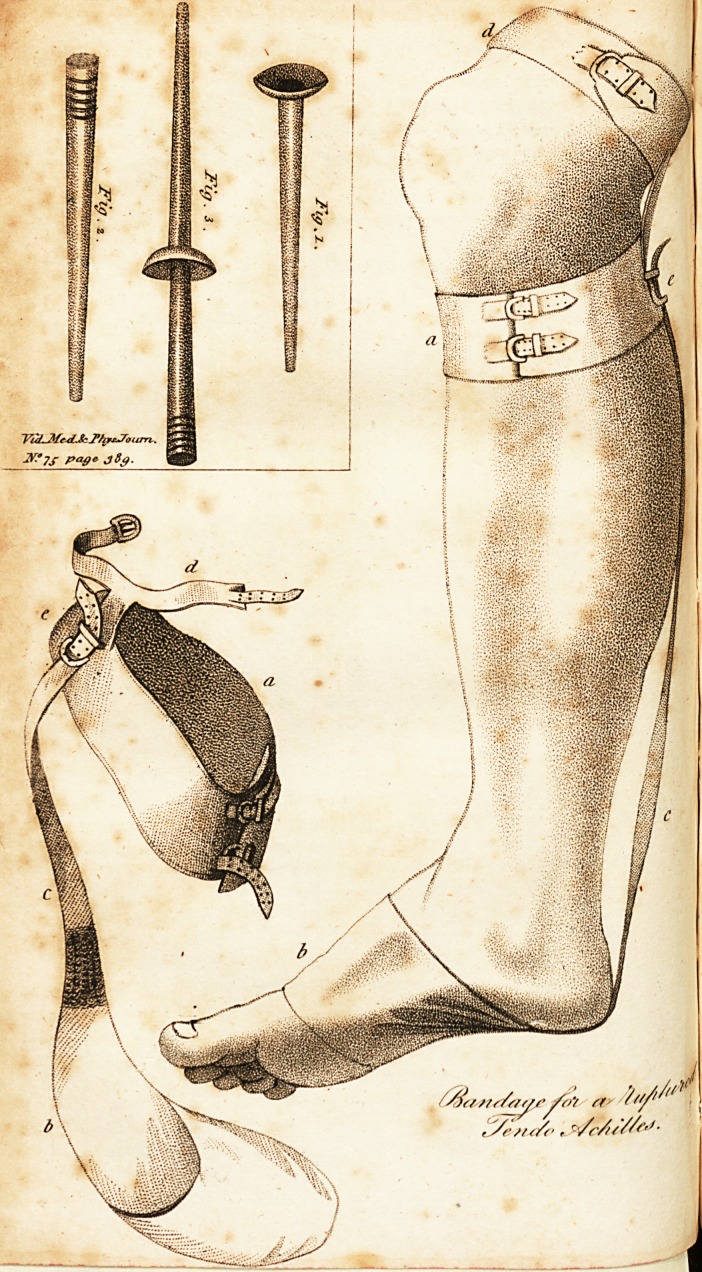# Mr. Waiblinger's Case of Ruptured Tendo Achillis

**Published:** 1805-06-01

**Authors:** J. Waiblinger

**Affiliations:** Member of the Royal College of Surgeons in London. Fulneck, near Leeds


### *To the Editors of the Medical and Physical Journal.*

Gentlemen,

I DO not know of any branch in surgery, where there is more room for real and useful improvement, than in the article of bandages. Many unpleasant accidents are daily occurring, which might, if not really cured, be much relieved, and made comparatively comfortable to the patients labouring under them, if more attention was paid to this branch of the profession. I own much has been done of late; but I am certain much still remains to be done. I have, for several years back, had an idea of a bandage for that unpleasant accident the *rupture* of the tendo achillis; but not wishing to intrude myself upon the public before I had realized its utility, I have not sent it to you sooner.

In country practice, such as mine is, accidents are not wanting; the particular one in question I have before met with more than once, and used the best methods that I had been taught or read of; but the cure was by no means satisfactory, either to my patients or myself; and I know of several cases, which were under the care of eminent surgeons, where the patients, from the shortening and contraction of the muscle, were under the necessity of wearing a high-heeled shoe, or to walk entirely on the toe.

My idea was to form, if I may be allowed the expression, an artificial muscle and tendon, which, at the same time that it assisted the patient in walking, kept the ruptured ends of the tendon in as close contact as possible, which must facilitate their union, the great contraction of the muscles, which always takes place, being overcome by the action of the springs. I herewith send you a drawing of the bandage as applied, and the same in profile, which I hope to explain by the references below. I shall just give a short account of the case alluded to.

—— P. Esq. a gentleman of large fortune and consequence in this neighbourhood, in walking in his park after a shower of rain, the ground being soft and clayey, the sward shot from

Vid. Med. & Phys. Journ.
N.o 75 page 389.

Bandage for a Ruptured

Tendo Achilles.

from under his feet, and he was thrown down; with the assistance of his servants he was got into the house. On being sent for, I soon found that the tendo achillis was torn through. As considerable spasms in the muscles came on, I ordered an embrocation with lin. saponis, tinct. opii, &c. and rest. As soon as the inflammatory symptoms abated, I sent for Mr. Wm. Morrison, bandage and truss maker, and a most ingenious mechanic, and directed him to make the bandage of which I enclose a drawing. Its success is indubitable. My patient wore it about nine months, and can now use his leg as well as ever. He is sixty-nine years of age. After the first week he could walk with tolerable ease, and subsequently with little difficulty.

By the public papers, I find accidents of the kind have been rather unusually frequent. I own this has been an additional stimulus to me to send the drawing. I had meant it to be in time for the last month, but my engagements prevented it. I hope the explanation of the drawing will be sufficiently clear. If it is not, I shall be very ready to give any farther explanation. I am, &c.

J. WAIBLINGER,
Member of the Royal College
of Surgeons in London.

*Fulneck, near Leeds, April* 12, 1805.

---

### Explanation of the Drawing.

No. 1. Bandage as applied.

*a.* A strap 2½ inches broad, to go round the leg, below the knee, fastened with two buckles.

*b.* A shoe of ticken, which draws tight at the tarsus. N. B. A whole shoe cannot be borne, as the spring strap draws the shoe so much, that it causes great pain in the toes.

c. A strong strap, fastened to the shoe *b.* 14 inches in length, and contains five spiral springs of brass wire, about the thickness of a quill barrel each; when inactive, the springs are about 3½ inches long. This strap is fastened to *a.* by buckle at *e.* and may be made to act at pleasure, by tightening or slackening it.

*d.* A strap, 1½ inch broad, buckled above the knee, is fastened by a small back strap to *a.* This back strap contains also several small spiral springs. Its chief use is, to take off some of the pressure upon the gastrocnemic muscles, which would else be troublesome.

P.S. I had prepared some observations on Vaccination, but as the benefits of the vaccine infection are so notorious, I forbear troubling you with them, being convinced it must find its due estimation in spite of all opposition. I have within the last four years inoculated about 1200, and have every reason to be satisfied with it.

Case

## Figures and Tables

**Figure f1:**